# Sustainable and Environmentally Friendly Microwave Synthesis of Nano-Hydroxyapatite from Decarbonized Eggshells

**DOI:** 10.3390/ma17081832

**Published:** 2024-04-16

**Authors:** Morsi M. Mahmoud

**Affiliations:** 1 Mechanical Engineering Department, College of Engineering and Physics, King Fahd University of Petroleum and Minerals, Dhahran 31261, Saudi Arabia; morsimahmoud@kfupm.edu.sa; Tel.: +966-533972633; 2 Interdisciplinary Research Center for Advanced Materials, King Fahd University of Petroleum & Minerals, Dhahran 31261, Saudi Arabia

**Keywords:** sustainability, waste management, eggshell decarbonization, microwave synthesis, nano-hydroxyapatite, rods-like crystals

## Abstract

The sustainable microwave (MW) synthesis of hydroxyapatite (HAp) from decarbonized eggshells was investigated. Decarbonization of eggshells, as a natural source of calcium carbonate (CaCO_3_), was carried out in the current study at ambient conditions to reduce the footprint of CO_2_ emissions on our environment where either calcination or acidic direct treatments of eggshells produce CO_2_ emissions, which is a major cause for global warming. Eggshell decarbonization was carried out via the chemical reaction with sodium hydroxide (NaOH) alkaline solution in order to convert eggshell waste into calcium hydroxide (Ca(OH)_2_) and simultaneously store CO_2_ as a sodium carbonate (Na_2_CO_3_) by-product which is an essential material in many industrial sectors. The produced Ca(OH)_2_ was mixed with ammonium dihydrogen phosphate (NH_4_H_2_PO_4_) reagent at pH~11 before being subjected to MW irradiation at 2.45 GHz frequency for 5 min using 800 Watts to prepare HAp. The prepared Nano-HAp was characterized using X-ray diffraction (XRD) where the crystal size was ~28 nm using the Scherrer equation. The elongated rod-like nano-HAp crystals were characterized using scanning electron microscopy (SEM) equipped with dispersive energy X-ray spectroscopy (EDS), Fourier transform infrared spectroscopy (FTIR), and transmission electron microscopy (TEM). MW synthesis of decarbonized eggshells is considered as a sustainable and environmentally friendly route to produce promising bioceramics such as nano-HAp. Concurrently, decarbonization of eggshells offers the ability to store CO_2_ as a high value-added Na_2_CO_3_ material.

## 1. Introduction

Waste management and sustainability should be considered in all humankind activities due to their associated environmental and economic impacts. A variety of natural waste materials should be utilized more effectively via sustainable waste management routes. While it may seem that some of these waste materials are useless at certain stages, their unexploited potential application is being revealed later. Nowadays, more attention is given toward waste management in order to convert generated wastes into useful materials to avoid its associated environmental side effects. On the other side, sustainable development will be greatly enhanced via converting those wastes into high value-added products. Eggshell waste is considered as one of the most common food wastes. In 2021, it was reported that around 250,000 tons of eggshells was formed annually all over the world [1]. This worldwide reported number is expected to increase dramatically in the near future where the consumption trend of chicken eggs as relatively affordable sources of protein is going to dramatically increase its waste quantity. The significant challenges associated with that massive waste generation will be directly affecting the environment and as a result the economy. Eggshell waste material usually turns into landfill or to a low value-added material. Eggshells are mainly calcite (calcium carbonate) with about 94 wt.%. Other components of eggshells, in wt.%, could be magnesium carbonate (1%), calcium phosphate (1%) and organic debris (4%) [2]. Remarkably, eggshell waste is considered as a hazardous waste according to European Union regulations [3]. Therefore, it is of great interest to convert eggshell waste into valuable bioceramic materials such as HAp for different applications [4]. The utilization of eggshells to produce HAp bioceramics materials was investigated extensively over the years using different routes [5,6,7] via either direct calcination, acidic or alkaline treatments of eggshells without decarbonization. It is expected that such utilization will not only reduce such waste but will also convert it into a useful HAp material.

**Figure 1:**
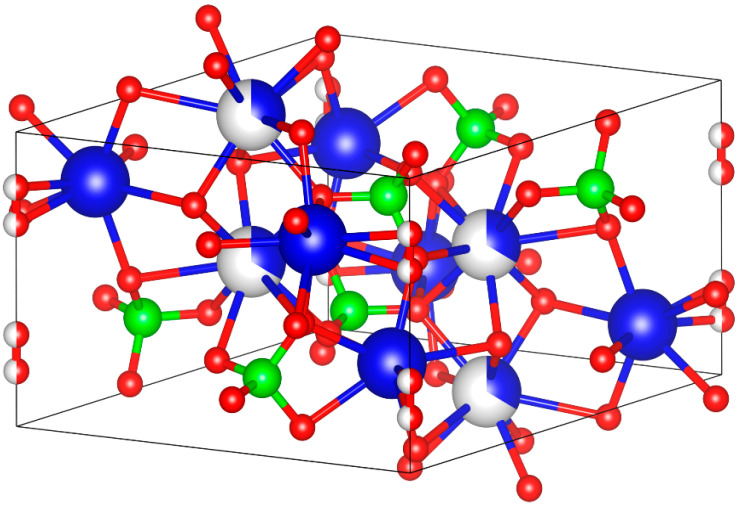
Hydroxyapatite unit cell (Ca: blue; P: green; O: red).

HAp is one of the most important biomaterials due to its similar structure to human bones and teeth as well as its unique ability to be integrated with tissues. It is an attractive material for many biomedical applications such as bone substitute materials in orthopedics and dentistry due to its excellent biocompatibility, bioactivity and osteoconduction properties [8,9,10,11]. HAp is an inorganic component found naturally in human hard tissues and also in other sources such as fish bones, coral, eggshells, chicken bones, etc. [12]. Stoichiometric HAp has the molecular formula of Ca_5_(PO_4_)_3_OH, which has a hexagonal dipyramidal crystal structure with a space group P6_3/m_ as shown in Figure 1 with the lattice parameters and unit cell volume shown in Table 1, where its unit cell has two formula units, so it is usually written as Ca_10_(PO_4_)_6_(OH)_2_ [13]. In the shown HAp unit cell, four Ca atoms (blue and white) are surrounded by nine O atoms from the phosphate moieties, while the other six Ca atoms (blue only) are surrounded by the other six O atoms from the phosphate moieties. HAp can include other traces such as phosphite ions (PO_3_^3−^), chloride ions (Cl^−^), fluoride ions (F^−^) and hydroxyl ions (OH^−^) depending on its source [8].

Microwave (MW) energy had been used as a promising processing tool for many materials because of its several advantages when compared to the commonly used traditional processing techniques [14,15]. MW is a powerful and significantly different tool to process several different materials with different applications where in most cases an improvement in the materials performance and properties were observed [16,17,18,19,20,21,22]. MW processing can lead to a short processing time, less energy consumption, selective heating and self-limiting reactions.

The circular economy is based on sustainability, recyclability and benefiting from waste materials via converting them into high value-added materials in a closed loop where the “3R” principles (reduce, reuse and recycle) are perfectly implemented. Based on that, waste should be turned into new products which consequently will lead to a significant increase in the sustainable economic development. Hence, industrial-scale conversion of eggshells waste into HAp material is expected to yield a higher economic value when compared with the expenses associated with the regular removal processes of such waste [23]. At the same time, this conversion will decrease in the danger of pathogen spread and will reduce the dumping costs. Hence, this approach will not only enhance the environmental profits but also will help greatly to achieve the ultimate goal of sustainable future and life.

The primary goals of this work are to decarbonize eggshell waste under ambient conditions via alkaline treatment with high conversion yield and to convert the resulting decarbonized eggshells precursor (Ca(OH)_2_) into a high value-added HAp material using microwave processing. Consequently, the 3R concept can be effectively implemented for a better sustainable future and life. To the best of our knowledge, almost all the reported previous studies to produce HAp from eggshells waste using different methods had never used the decarbonizations route for eggshells. Furthermore, this new proposed eggshell decarbonization route is going to safely contain and store CO_2_ as a valuable sodium carbonate byproduct, soda ash, that can be used in different useful industrial sectors which makes the whole process more environmentally and economically appealing.

## 2. Materials and Methods

Eggshell waste material was collected from KFUPM food court restaurant where it was subjected to tap water washing several times before it was later dried at 110 °C for 3 h in an oven. The dried collected eggshells waste was then crushed in an electric motor blender for 30 min before it was sieved and passed through a 100-mesh sieve (~149 µm) to give a homogenous powder. The resulting prepared powder was then used for the decarbonization route where specific amounts of eggshell powder were mixed with sodium hydroxide pellets (from Sigma Aldrich, Burlington, MA, USA) and distilled water as shown in Table 2 to achieve a maximum conversion percentage of ~96% of eggshell waste into calcium hydroxide (Ca(OH)_2_) and sodium carbonate (Na_2_CO_3_), as the resulting materials from this decarbonization process, as per the following chemical reaction:

(Eggshell) CaCO_3_(s) + 2NaOH(aq) + xH_2_O → Ca(OH)_2_ + Na_2_CO_3_·xH_2_O (x = 0 or 1)

The previous parameters, shown in Table 2, were adapted from the study carried out by Hanein et al. [24] where the decarbonization of commercial CaCO_3_ at atmospheric temperatures and pressures were investigated and described in more detail in which different starting proportions of CaCO_3_, NaOH and H_2_O were studied to achieve different sufficient conversion percentages.

**Figure 2:**
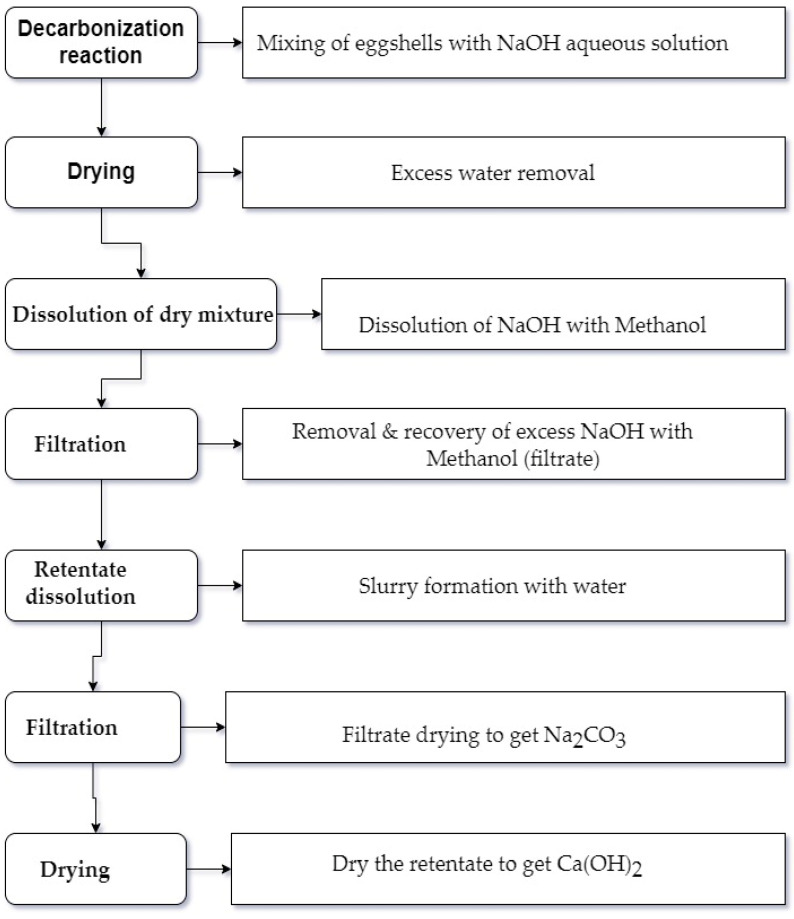
Schematic chart for eggshell decarbonization process to obtain Na_2_CO_3_ and Ca(OH)_2_.

In the current study, the decarbonization reaction was accomplished under ambient conditions, using Table 2 parameters, with continuous mixing using a magnetic stirrer in a 100 mL Teflon beaker for ~10 min. A vacuum filtration system was used to separate the products via different steps. The unreacted excess NaOH was recovered using methanol alcohol from the other compounds. Later, with water preferential dissolution, the other two resulting precipitates (Ca (OH)_2_ and Na_2_CO_3_) were obtained and dried separately as shown in Figure 2. The obtained dried sodium carbonate, soda ash, was then stored in a closed container for future usage while the other obtained dried calcium hydroxide powder was used as the calcium source precursor for the HAp synthesis using microwave processing.

Mixing of 300 mL Ca(OH)_2_ solution obtained from the ambient condition decarbonization process along with 100 mL solution of ammonium dihydrogen phosphate (NH_4_H_2_PO_4_) reagent (Sigma Aldrich), as the phosphate source, was carried out drop wise with continuous stirring to maintain the resulting mixture pH at around 11 using the Orion Star A215 pH meter. The concentration of Ca^2+^ and PO_4_^−3^ in the mixture was 0.7775 and 0.4656 M, respectively, which correspond to the Ca/P molar ratio of 1.67, as the theoretical molar ratio of HAp, according to the following reaction:

10Ca(OH)_2_ + 6NH_4_H_2_PO_4_ → Ca_10_(PO_4_)_6_(OH)_2_ + 6NH_4_OH + 12H_2_O

**Figure 3:**
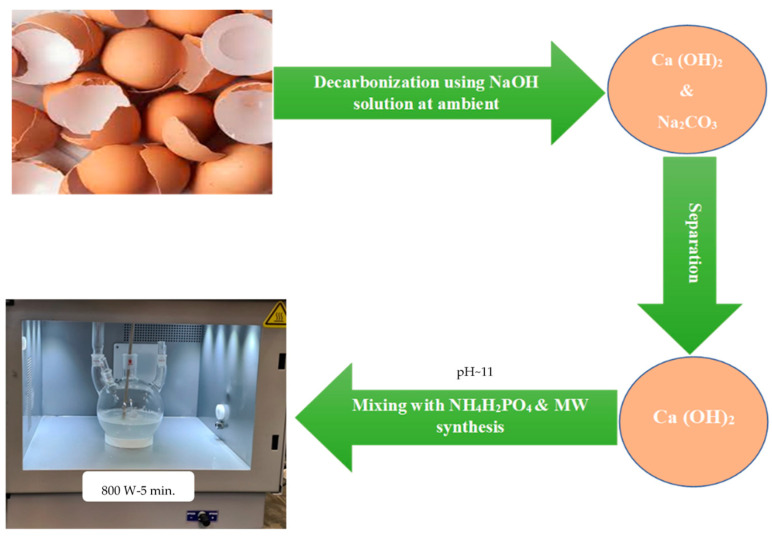
Microwave synthesis setup of HAp from decarbonized eggshell.

Later, the resulting mixture was transferred into a 4-neck flask and directly heated using the MW synthesis unit, Sineo MAS-II Plus (Sineo Microwave Chemistry Technology Co., Ltd., Shanghai, China), where the mixture was exposed to MW radiation at 2.45 GHz for 5 min using 800 Watt. The temperature was controlled at around 95 °C with continuous stirring during MW exposure time, as shown in Figure 3. After cooling, the reaction mixture was collected, filtered through vacuum filtration system, and washed several times with distilled water before being dried in a conventional oven at 110 °C for 3 h. The obtained dried Hap powder was characterized using X-ray diffraction (XRD) with Rigaku Miniflex-II Mini-X-ray Diffraction machine (Rigaku, Tokyo, Japan) at 2° min^−1^ rate using Cu K_α_ source with 0.15406 nm wavelength, where a 10 mA current and 30 kV voltage were used. Furthermore, the two resulting precipitates, Ca (OH)_2_ and Na_2_CO_3_, were also characterized using XRD to confirm the successful completion of the decarbonization process under ambient conditions. FTIR analysis was performed on a Smart iTR NICOLET iS10 FTIR machine (Thermo Fisher Scientific, Waltham, MA, USA). Wavenumber 4000 cm^−1^ to 400 cm^−1^ was scanned with a resolution of 4 cm^−1^ and 16 scans. Furthermore, the HAp powder was also characterized using a Field emission scanning electron microscopy (FE-SEM) model (FEI Quanta 250) equipped with an EDS detector (150 mm^2^ Oxford detector) with accelerating voltage 200 V–30 kV, where the investigated powders were platinum-coated using a Quorum coating machine model Q150R (Quorum Technologies, Lewes, UK). A JEM-2100F field emission electron microscope (FE TEM) (JEOL, Tokyo, Japan) with 200 kV was used to investigate the obtained HAp powder where the powder was dispersed in 2-propanol/water and sonicated before testing.

## 3. Results

**Figure 4:**
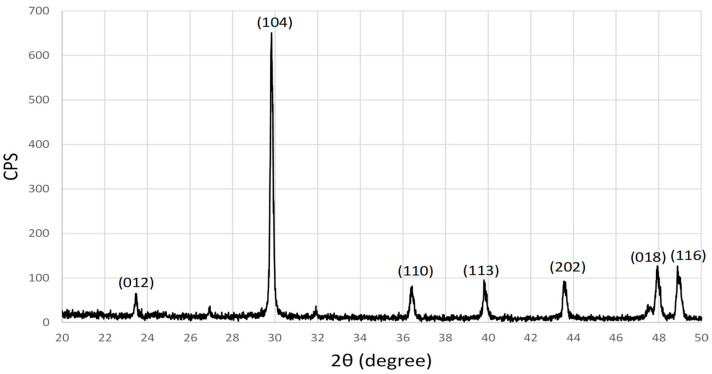
XRD of the eggshell powder.

Figure 4 shows the XRD pattern of eggshell powder after being cleaned, crushed and sieved. The XRD peaks were indexed and identified as CaCO_3_, as expected, where the peaks match with the Joint Committee on Powder Diffraction Standards (JCPDS) file No. 05-0586 [25]. The crystallite size of the eggshell powder was calculated to be around 61 nm for (104) planes using the Scherrer equation as shown below:

$$\beta =\frac{k\;\lambda }{L\cos\;\theta }$$

where β is the full-width at half-maximum (FWHM) of the diffraction peak in radians, L is crystalline size in (Å), k = 0.9, θ is the Bragg angle, and λ = 1.5406 Å.

**Figure 5:**
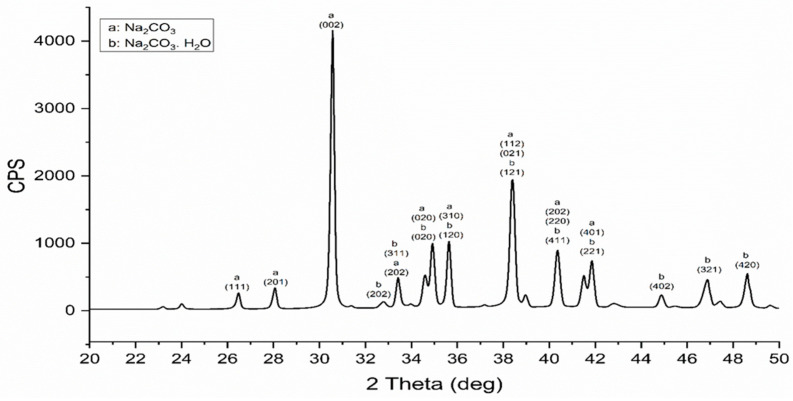
XRD of sodium carbonate mixture by-products resulted from the decarbonization process.

The XRD of the sodium carbonate by-product that was formed after the decarbonization process of eggshell is shown in Figure 5. The XRD analysis shows that the major peaks are matching very well with sodium carbonate compounds where a mixture of sodium carbonate (NaCO_3_) and hydrate sodium carbonate (Na_2_CO_3_·H_2_O) are formed [26]. The detected sodium carbonate (NaCO_3_) by-product is donated by the letter (a) in the XRD pattern with a calculated crystal size of around 48 nm for the (002) plane. It is identified as a monoclinic crystal system with space group (P21/n) where its peaks are indexed according to the card # c11-1130 of JCPDS—International Center for Diffraction Data (ICDD) [27]. On the other hand, the hydrate sodium carbonate (Na_2_CO_3_·H_2_O) is donated by the letter (b) in the graph with a crystal size of 42 nm for the (420) plane. Moreover, it is indexed as an orthorhombic-crystal-structure monoclinic crystal system with a space group (Pca21). Its peaks are indexed according to the card # c08-0448 of JCPDS data [28]. This confirms the successful decarbonization of eggshells without any release of carbon dioxide and simultaneously storing CO_2_ as useful soda ash material.

**Figure 6:**
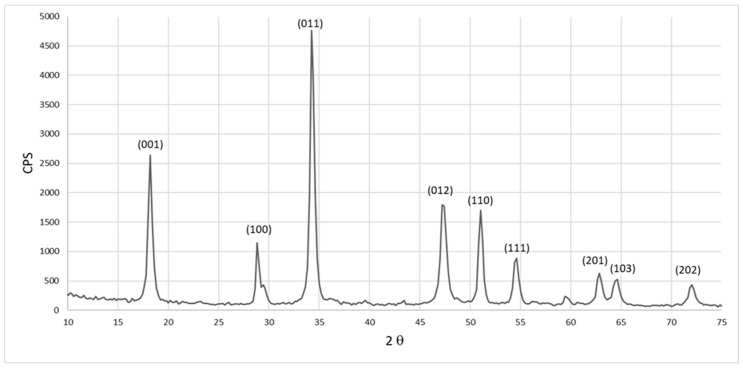
XRD of calcium hydroxide by-product resulted from the decarbonization process.

On the other hand, the XRD pattern of the resulting calcium hydroxide by-product, Ca(OH)_2_, that was also formed as a results of the decarbonization process of the eggshell is shown in Figure 6. This formed calcium hydroxide (Ca(OH)_2_) by-product is identified as a hexagonal crystal system with space group (P-3m1) with calculated crystal size of around 15 nm for (011) plane. The detected peaks are indexed according to JCPDS file number (01-073-5492) and agree with previous work [29]. This proves the successful formation of the Ca(OH)_2_ by-product from the decarbonization process of eggshell waste that will be used as the main source of calcium for HAp synthesis using MW.

**Figure 7:**
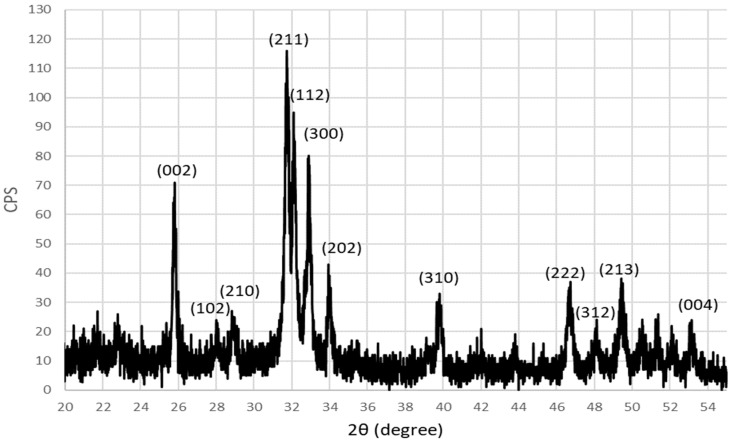
XRD pattern of microwave-synthesized HAp using decarbonized eggshell.

The XRD pattern of microwave-synthesized HAp powder using decarbonized eggshells is shown in Figure 7, where the 2θ scan range was between 20° to 55°. The existence of the unique intense peak at 2θ = 31.75° for the (211) plane is identified, which is a characteristic peak for the apatite formation, in addition to the other remaining peaks that are related to the HAp structure. So, the XRD pattern indicates the successful formation of a single pure crystalline hexagonal HAp phase where it is indexed and identified in accordance with JCPDS file number (09–0432) and matches well with the literature [19,30,31]. The calculated crystal size for synthesized HAp was found to be around 28 nm, using the Scherrer equation on the (002) plane. So, MW synthesis of the decarbonized eggshell had successfully produced pure nano-sized HAp as revealed by the XRD pattern.

**Figure 8:**
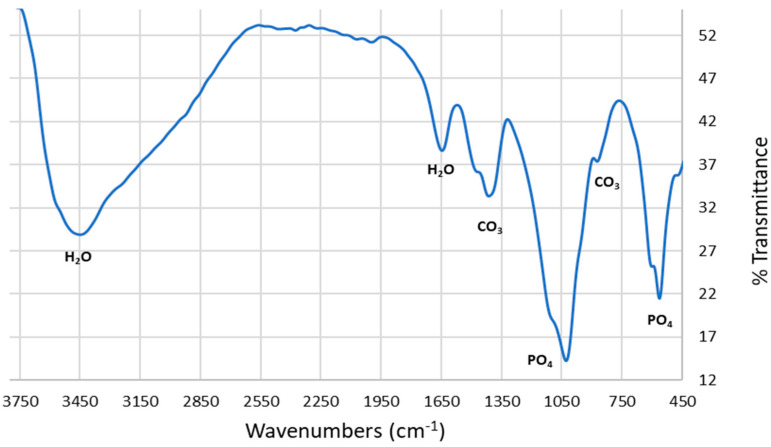
FTIR spectrum of microwave-synthesized HAp using decarbonized eggshell.

The FTIR spectra of the synthesized HAp is shown in Figure 8. The absorption bands at around 1430 and 867 cm^−1^ are assigned to the presence of carbonate ions in the HAp sample which is due to the reaction of atmospheric carbon dioxide with HAp during synthesis, while the unique [PO_4_]^−3^ stretching mode is observed at 1022 cm^−1^. Furthermore, the other bending modes of [PO_4_]^−3^ is observed at around 565 and 585 cm^−1^. The peaks around 1640 and 3430 cm^−1^ are due to the vibration modes of the H_2_O molecule which matches what was reported in the literature [8,32,33,34].

**Figure 9:**
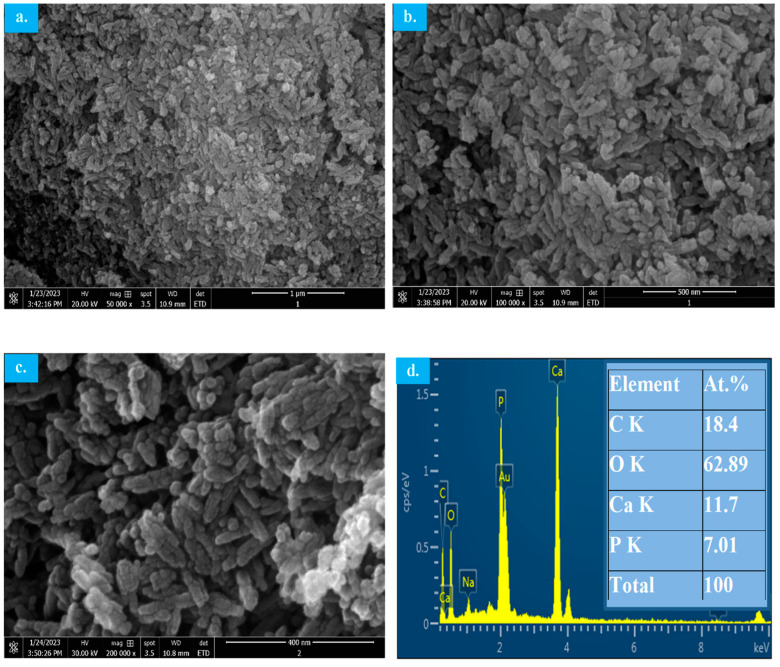
SEM-EDX micrographs of MW-synthesized HAp using decarbonized eggshell. (**a**–**c**) SEM micrographs; (**d**) EDX.

SEM micrographs of the MW-synthesized HAp from the decarbonized eggshell are shown in Figure 9. The morphology of the synthesized HAp appears to be an agglomeration of individual particles with rod/needle-like shapes in the nano-size range as predicated from the Sheerer equation using the XRD pattern. The individual particles’ exact shape could not be easily recognized from SEM micrographs due to their tiny nano-size. The EDX analysis is shown in Figure 9d where the peaks of calcium, phosphorous and oxygen indicate the synthesis of HAP with the exact Ca/P ratio of 1.67 as designed in the synthesis step.

**Figure 10:**
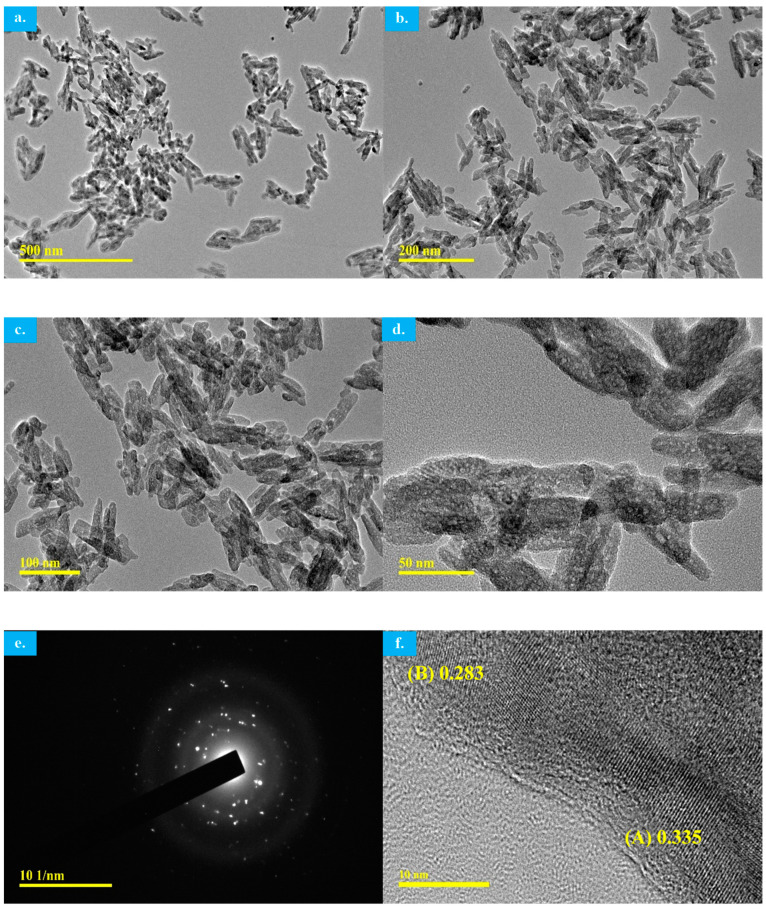
(**a**–**f**) TEM images of the MW-synthesized HAp using decarbonized eggshell.

TEM images of the MW-synthesized HAp are shown in Figure 10. The figure reveals a highly crystalline apatite structure without the presence of any other crystalline phases that is consistent with the XRD and FTIR analyses. TEM images clearly show the HAp particle shape as it is now much easier to define as highly agglomerated and elongated nano-rods/needles with an average size of around 25 ± 5 nm. The selected-area electron diffraction (SAED) pattern (Figure 10e) exhibits concentric rings that represent the presence of the polycrystalline structure of MW-synthesized HAp which is in agreement with the XRD pattern [8].

The interplanar lattice fringe spacing of the MW synthesized HAp using decarbonized eggshell were measured as in Figure 10f. At point A, the d spacing is calculated to be 0.335 which corresponds to the (0002) plane, the middle basal plane of the HAp hexagonal unit cell. This is actually in closer agreement with an earlier study investigating an apatite hexagonal cell structure where the d-spacing value of 0.34 nm for the same plane was reported [35]. Furthermore, the d spacings for the (002) plane of the crystalline particles was reported to be 0.34 nm in another investigation for a needle-shaped apatite crystal structure [36]. Point B in Figure 10f shows a measured value of 0.283 nm that closely corresponds to the figure of the lattice plane, which is also in close agreement with the same earlier study [35] where they reported d-spacings of 0.281 nm for that same lattice plane of a hexagonal apatite cell. Furthermore, the d spacing between lattice fringes of needle-shaped apatite crystal plate particles at (211) or (112) planes, the two major XRD peaks for HAp, were reported to be in the range of 0.27–0.29 nm [36] which is a closer match to the measured point B value in Figure 10f of the MW-synthesized nano-sized HAp.

## 4. Discussion

### 4.1. Decarbonization of Eggshells Waste

First, if eggshell waste has been treated via either direct calcination or direct acidic treatment, as in most of the reported works [1,3,37], to produce HAp materials from such waste, there will be a release of greenhouse emission gas (CO_2_) from both routes. These emissions will have a direct negative effect on the environment where the amount of greenhouse emissions is going to increase. Detailed calculations for the amount of CO_2_ emissions from both methods are provided below.

#### 4.1.1. Direct Calcination of Eggshells

The detailed equation with molar mass calculation for that calcination route is shown below where it can be concluded that if around 100 g of eggshells, CaCO_3_, will be calcined, it will produce around 56.1 g of calcium oxide (CaO), which will be used as the Ca precursor for HAp preparation. On the other hand, the amount of CO_2_ emissions released from that direct calcination of eggshells method will be around 44 g.

CaCO_3_ (s) = CaO (s) + CO_2_ (g)

(100.1 g) (56.1 g) (44 g)

#### 4.1.2. Acidic Treatment of Eggshells

Furthermore, almost a similar amount of CO_2_ emissions will be released to the environment if eggshells are directly treated with acids such as hydrochloric acid (HCl), as an example and as per the equation below, assuming an excess of HCl where the reaction will run until the CaCO_3_ is totally used up. So, the acidic treatment route of eggshells will also produce around 44.0 g (around 22.414 L) of CO_2_, if around 100.1 g of eggshells powder is treated with acids.

CaCO_3_ (s) + 2HCl (aq) = CaCl_2_ (s) + CO_2_ (g) + H_2_O (aq)

  (100.1 g)  (72.9 g)   (111.0 g)   (44.0 g)    (18.0 g). 

In conclusion, assuming using any of the two above discussed methods, if one (1) ton/day of eggshells is processed to produce HAp via either calcination or acidic treatment, CO_2_ gas emissions of around 440,000 g/day (440 kg/day) will be released to our environment. Consequently, both routes of HAp production will have a direct negative impact on our environment and will increase the greenhouse emissions. Its negative effects are similar to consuming around 1 barrel of oil/day, or similar to burning around 187.4 L of gasoline, or similar to burning around 220 Kg of coal/day. These previous terms and metrics were determined according to the calculations estimated using the “Greenhouse Gas Equivalencies Calculator” managed by the Environmental Protection Agency (EPA), Washington, DC, USA [38]. This online calculator tool transforms emissions data into easily comprehensible terms. With the use of this calculator, you may convert intangible data into easily understood metrics. This calculator could be very helpful when explaining a given greenhouse gas reduction plan, reduction objectives, or other actions focused on lowering greenhouse gas emissions. It is based on solid calculations and conversion factors from scientific work, data and research. As an example of those greenhouse amount calculations for one of the previously mentioned metrics, which is a barrel of oil consumed, the CO_2_ emissions/barrel of crude oil are calculated by the calculation example below via the multiplying of the average heat content from 1 barrel times the carbon coefficient times the fraction oxidized times the ratio (44/12) of the molecular weight of CO_2_ to that of carbon.

Ex: 5.80 mmbtu/barrel × 20.31 kg C/mmbtu × 44 kg CO_2_/12 kg C × 1 metric ton/1000 kg = 0.43 metric tons CO_2_/barrel

where the average heat content of crude oil is 5.80 million British thermal units (mmbtu) per barrel [39]. The average carbon coefficient of crude oil is 20.31 kg carbon per mmbtu [39]. The fraction oxidized is assumed to be 100 percent [40].

#### 4.1.3. Decarbonization of Eggshells with NaOH

In the current work, at room temperature, the decarbonization of eggshells is carried out using NaOH to obtain Ca(OH)_2_, which will be used later as the calcium precursor for the HAp preparation. Furthermore, calcium carbonate by-products are also formed from that decarbonization process where the CO_2_ emissions are not going to occur and are actually sequestered due to the formation of that by-product, as shown in the two equations below:

10CaCO_3_(s) + 20NaOH(aq) + xH_2_O = 10Ca(OH)_2_ (s) + 10Na_2_CO_3_·xH_2_O (x = 0 or 1) (s)

(1000.9 g)   (799.9 g)      (740.9 g)     (1059.9 g)      

10Ca(OH)_2_ (s) + 6NH_4_H_2_PO_4_ (s) = Ca_10_(PO_4_)_6_(OH)_2_ (s) + 6NH_4_OH (aq) + 12H_2_O (aq)

(740.9 g)    (690.2 g)    (1004.6 g)       (210.3 g)    (216.2 g)

According to the molar mass calculation of the above equations, if around 1 kg of eggshell waste is decarbonized by the addition of 799.9 g of sodium hydroxide, it will produce 740.9 g of calcium hydroxide and 1059.9 g of sodium carbonate Na_2_CO_3_. The formation of Na_2_CO_3_ is going to store and sequestrate around 440 Kg of CO_2_ emissions from being released to the environment. On the other hand, that formed amount (740.9 g) of calcium hydroxide will be used to synthesize around 1 kg of the value-added HAp product after the addition of ammonium dihydrogen phosphate using microwave energy processing.

Consequently, this decarbonization route will have a positive effect on the environment and is considered as a sustainable and green synthesis route where if only one (1) ton of HAp is produced/day from that decarbonized eggshells waste route, its positive environmental effect is equivalent to carbon sequestered by around 7.3 tree seedlings grown for 10 years based on that 1 ton/day calculations.

Furthermore, if we extrapolate this HAp production process using this decarbonization route for a year (i.e., 1 ton of HAp for 365 days), this will be equivalent to carbon sequestered by around 2656 tree seedlings grown for 10 years, or similar to avoiding CO_2_ emissions from around 68,406 L of gasoline consumed, or similar to avoiding greenhouse emissions from around 80,283 Kg of coal burned, or similar to avoiding greenhouse emissions resulting from consuming around 372 barrels of oil. These previously discussed positive effects on the environment will be added to the expected economic gain via converting this hazard eggshell waste into high value-added products such as hydroxyapatite and soda ash that are actually used in many applications and fields.

### 4.2. Microwave-Assisted Synthesis of HAp from Decarbonized Eggshells

As discussed before, the decarbonized eggshell by-product Ca(OH)_2_, which is the calcium source, was effectively reacted with NH_4_H_2_PO_4_ to create nano-HAp via MW synthesis. In less time, such as 5 min only, a single phase of pure nano-sized HAp biomaterial with a crystal size of 28 nm was produced. XRD, FTIR and electron microscopy (SEM-EDX and TEM) were used to analyze the synthesized nano-HAp. XRD examination revealed the production of HAP and provided the distinctive apatite peak for 2θ values between 31.9 and 32.2. Furthermore, the FTIR spectra’s peaks show that this HAp MW-synthesized sample contains hydroxyl (OH^−^), phosphate (PO_4_^3−^) and carbonate (CO_3_^2−^) groups, indicating that the HAp was synthesized successfully. An aggregation of individual particles with rod-like particles was seen using SEM and TEM, and they were recognized as part of the distinct polycrystalline hexagonal crystal structure of HAp. With its distinct Ca/P ratio of 1.67, EDX data have verified the synthesis of HAp. FE-TEM was used to measure and identify the d-spacings of the principal lattice planes in the hexagonal HAp lattice cell and its particles. In general, the crystal size, structure and the morphology of the crystals of the MW-synthesized Hap from the decarbonized eggshell source is comparable with what was reported in the literature [1,19,30], confirming the successful preparation of HAp from that decarbonized eggshell waste.

## 5. Conclusions

The decarbonization process of eggshell waste was achieved successfully with a high conversion percentage using the alkaline sodium hydroxide solution treatment resulting in two (2) valuable by-products. The first one was calcium hydroxide that was used as a calcium source precursor for nano-HAp preparation using MW synthesis, and the second one was soda ash (Na_2_CO_3_). The decarbonization process will not only transfer this waste into valuable biomaterial such as nano-HAp but also will store and trap carbon dioxide as a major global warming and climate change factor into a useful by-product, soda ash (Na_2_CO_3_). In fact, soda ash global reserves are really limited and not geographically widespread, while it is being used in vast industrial activities such as ceramics, glasses, detergents and chemicals industries. This process of eggshell decarbonization is economically and environmentally appealing since almost 1 mole of CaCO_3_ is going to produce an almost equal amount of soda ash and the Ca(OH)_2_ precursor that is going to be converted later into nano-sized HAp material, as a promising biomaterial material. The decarbonization process is vital for such eggshells hazard waste, as defined by European union regulation where it will satisfy the “3R” concept of circular economy and sustainability demand for better human life. This decarbonization route is having several positive environmental metrics and effects on lowering the greenhouse emissions for better green and sustainable recycling of eggshell waste. Furthermore, MW synthesis was successfully used to prepare nano-HAp via the reaction of the decarbonized eggshell by-product, Ca(OH)_2_ with NH_4_H_2_PO_4_. A single phase of pure nano-sized HAp biomaterial with a crystal size of 28 nm was prepared in a shorter time (5 min). The prepared nano-HAp was characterized using XRD, FTIR and electron microscopy (SEM-EDX and TEM), where an agglomeration of individual particles with rod-like particles were observed and identified with their unique polycrystalline hexagonal crystal structure of HAp. EDX data had also confirmed the formation of HAp with its unique Ca/P ratio of 1.67. The d-spacings of the major lattice planes in the hexagonal HAp lattice cell and its particles were measured and identified using FE-TEM.

## Figures and Tables

**Table 1:** Crystal structure and lattice parameters of HAp [13].

Crystal System	Space Group	a (A)	b (A)	c (A)	α	β	γ	Unit Cell Volume (A3)
Hexagonal	P6_3/m_	9.41898	9.41898	6.88119	90°	90°	120°	528.6910

**Table 2:** Used eggshell decarbonization parameters with 96% conversion.

Precursors	Eggshells	NaOH	H_2_O	Conversion (%) to Ca(OH)_2_
Amount in (g)	1.00	4.61	6.75	96%
wt.% [24]	8.1	37.3	54.6	

## Data Availability

All data generated or analyzed during this study are included in this published article.
